# The 2008 Financial Crisis and Changes in Lifestyle-Related Behaviors in Italy, Greece, Spain, and Portugal: A Systematic Review

**DOI:** 10.3390/ijerph18168734

**Published:** 2021-08-18

**Authors:** Monica Sane Schepisi, Anteo Di Napoli, Rosario Asciutto, Simona Vecchi, Concetta Mirisola, Alessio Petrelli

**Affiliations:** 1 National Institute for Health, Migration and Poverty (INMP), 00153 Rome, Italy; msaneschepisi@gmail.com (M.S.S.); anteo.dinapoli@inmp.it (A.D.N.); rosarioasciutto@gmail.com (R.A.); concetta.mirisola@inmp.it (C.M.); 2 Ministry of Health—General Directorate for Health Prevention, 00144 Rome, Italy; 3 Department of Epidemiology—Lazio Region, ASL Rome 1, 00147 Rome, Italy; s.vecchi@deplazio.it

**Keywords:** economic crisis, COVID-19, risk behaviors, lifestyles, inequalities, socioeconomic

## Abstract

Italy, Greece, Spain, and Portugal have all been strongly affected by the 2008 financial crisis, which has had a negative impact on health. We systematically evaluated the effects of the crisis on lifestyle and socioeconomic inequalities. We conducted a literature search using MEDLINE, Embase, the Cochrane Library, and health economics databases for studies reporting quantitative comparisons before and after (or during) the crisis on the following risk behaviors: alcohol consumption, smoking habit, healthy diet, physical activity, and psychotropic drugs and substance abuse, without setting any age restrictions. We selected 34 original articles published between 2011 and 2020. During/after the crisis, alcohol consumption and substance abuse decreased, while psychotropic drug use increased. We also observed a deterioration in healthy eating behavior, with a reduction in fruit and vegetable consumption. Smoking habit and physical activity showed a more complex, controversial trend. Socioeconomic inequalities were affected by the recession, and the negative effects on unhealthy lifestyle tended to be more pronounced among the disadvantaged. These results suggest the need to implement health policies and interventions aimed at monitoring risk behaviors, with special regard to disadvantaged people, and considering the potential additional impact of the COVID-19 pandemic.

## 1. Introduction

Before the COVID-19 pandemic, the 2008 Great Recession was the most severe crisis experienced by Europe since the Second World War, particularly in the southern European countries of Italy, Greece, Spain, and Portugal [1]. The considerable amount of literature on the topic shows that the financial crisis has had a strong impact on the health of most European populations [2]. Despite the trend towards reduced mortality, a deterioration in mental health, an increase in the number of suicides and, to a varying extent, in some non-communicable and communicable diseases, and a worsening in perceived health has been observed in most European populations [3,4]. However, an increase in mortality due to alcohol-related causes and to the consumption of drugs [5] has been observed in some countries.

Italy, Greece, Spain, and Portugal have been affected both by the direct effects of the financial crisis on the health of their populations and by the barriers to healthcare access imposed by the austerity policies introduced by governments to pay off the public debt [2]. In Greece, the austerity measures implemented to contain public spending contributed to an increase in forgoing health care due to economic reasons, especially among the poor, people with lower incomes, and the unemployed [6]; similar results were observed in Italy [7]. A deeper analysis by Karanikolos [2] suggests that, although recession poses risks to health, the interaction between fiscal austerity with economic shocks and weak social protection is what ultimately seems to escalate health and social crises in Europe.

The impact of the financial crisis on the health of the populations has been disproportionate. In fact, the more limited decrease in the number of deaths among the more disadvantaged social groups compared to the general population has determined a widening of mortality inequalities [8]. A recent systematic review has found an increase in socioeconomic inequalities [9]. Historical evidence supports the hypothesis that recession periods are associated with worse lifestyle, including increased alcohol consumption [10] or drug use [11], but the association between the Great Recession and worse lifestyle has not yet been systematically evaluated.

In this context, the COVID-19 pandemic began while the effects of the 2008 financial crisis were still manifesting; in addition to the dramatic impact on mortality and the direct long-term effects on the health of those who have recovered, there have also been indirect effects due to the cancellation or postponement of non-urgent assistance or interventions to decongest overwhelmed care facilities, technologies, and personnel. 

The purpose of our review was to systematically evaluate the effects of the 2008 financial crisis on lifestyle and socioeconomic inequalities in Italy, Greece, Spain, and Portugal. 

## 2. Materials and Methods

The review protocol was registered (CRD42019129105) in the PROSPERO open access database of systematic reviews (Available online: http://www.crd.york.ac.uk/PROSPERO) (accessed on 12 August 2021). Conducting and reporting are in accordance with PRISMA guidelines. The Covidence systematic review software (Veritas Health Innovation, Melbourne, VIC, Australia. Available online: http://www.covidence.org) (accessed on 12 August 2021) was used as the reference manager tool for the phases of importation, deduplication, and selection. 

### 2.1. Information Sources and Search Strategy

The literature search was performed using MEDLINE (via Ovid), Embase, The Cochrane Library and health economics databases (EconLit). Search terms for financial crisis were combined with the terms “eating behavior”, “smoking habit”, “alcohol consumption”, “psychotropic drug use”, “drug abuse”, or “gambling”. Supplementary Table S1 shows the full search strategy for MEDLINE. To identify additional relevant documents, the grey literature was searched for using OpenGrey and through the screening of the websites of the following referral organizations on population health and healthcare: The World Health Organization, the Organization for Economic Co-operation and Development, the European Observatory on Health Systems and Policies, the European Commission, and the European Centre for Disease Prevention and Control. 

The references of included articles were also screened to identify potentially eligible articles for inclusion. 

We included observational studies reporting quantitative comparisons before and after, before and during, or during and after the crisis of the following key health behaviors: diet, smoking, physical activity, alcohol consumption, and psychotropic drug use or substance abuse affecting individuals of any age. We considered studies published between January 2008 and November 2020 in English, Italian, Spanish, Portuguese, and Catalan. Multi-country studies were included when individual country data were available.

The exclusion criteria were:

1. Type of study: publications lacking primary data and/or explicit descriptions of the methods. Abstracts, editorials, correspondence, and commentaries were deemed acceptable for inclusion if they reported sufficient data;
2. Methodology: pre–post comparison missing;
3. Data not suitable for extraction (e.g., reporting data on a group of countries);
4. Study population overlap.

Studies were selected through a three-step selection procedure based on: (1) screening of title and abstract, (2) screening of full-text article, and (3) final screening during the data extraction phase. Two independent researchers with experience in reviews and in the topic filtered and selected the references. In cases of discordance, a third researcher was consulted to determine inclusion or exclusion of the reference.

### 2.2. Data Extraction, Quality Evaluation, and Synthesis of Results

The following data were extracted from each included article: study population (number, age range, sex), population characteristics (e.g., students, household members), data source (e.g., national registry, questionnaires), study design, outcome definition, results (if available, by sex and socioeconomic status), main conclusions.

Two independent researchers judged the quality of each eligible study using a modified Newcastle-Ottawa Scale (NOS) for cross-sectional studies [12]: a study is assigned a maximum total score (stars) of nine for the following domains: selection, comparability, and outcome. We considered scores of 0–3, 4–6, and 7–9 as indications of low, medium, and high quality, respectively. Any doubt was resolved by consulting with a third reviewer. The score of the included articles are shown in the Supplementary Table S2. 

The results, organized by country, report study population (number and age range) and data sources, outcome definition, results, and effects on inequalities, if reported. Given the highly heterogeneous nature of the studies, we did not attempt to conduct a meta-analysis, and we report the results narratively.

## 3. Results

**Figure 1:**
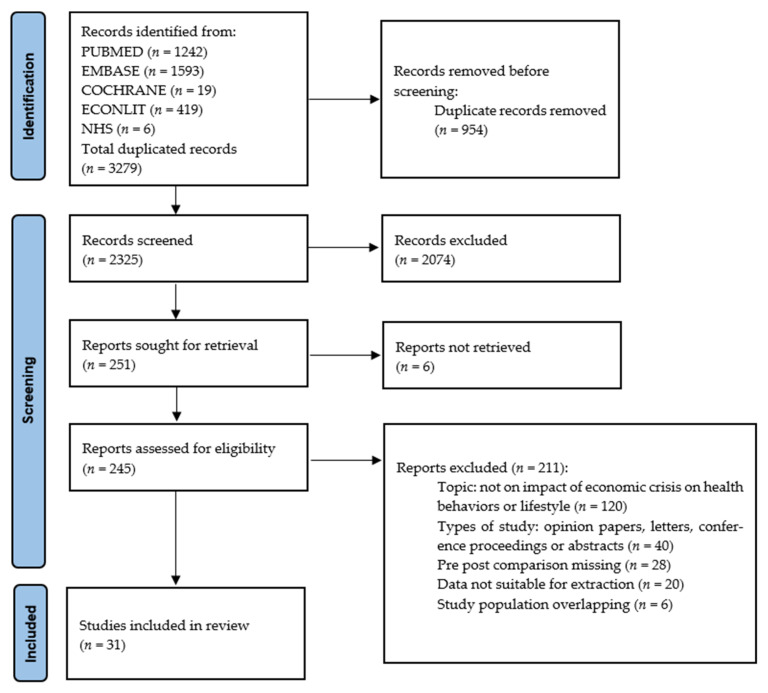
PRISMA flow diagram.

Our search identified 2325 unduplicated records; after the selection process, 31 cross-sectional studies [13,14,15,16,17,18,19,20,21,22,23,24,25,26,27,28,29,30,31,32,33,34,35,36,37,38,39,40,41,42,43] were identified, as detailed in the PRISMA flow diagram (Figure 1). The main characteristics of the eligible studies are reported in Table 1. The articles, published between 2011 and 2020, were conducted primarily in Spain (*n* = 19); the remainder were conducted in Italy (*n* = 6), Greece (*n* = 6), and Portugal (*n* = 4). Two studies were multi-country [19,34]. Of the studies included, six also considered a juvenile population (age ≤ 15 years) [21,29,33,34,41,43], while the remaining were on adolescents aged > 15 years and on adults. The studies used validated questionnaires or administrative registries as data sources. 

Regarding the outcomes considered, most of the studies concerned diet (*n* = 18) and smoking habit (*n* = 17), followed by alcohol consumption (*n* = 15) and physical activity (*n* = 13). Antidepressant/anxiolytic/antipsychotic drug use was examined by nine studies and substance abuse by six. In order to report briefly the main findings of the studies from a public health perspective, we created Table 2, which shows the variation in health behavior during or after the 2008 crisis by means a symbol for each risk behavior.

Regarding study quality, the available evidence was affected by a high risk of bias for exposure and for outcome assessment due to the study design, use of self-reported measures, and lack of adjusting for potential confounding factors. Supplementary Table S2 summarizes the NOS assessment of the included studies, which obtained scores between 2 and 7, with an average score of 4.7; four studies appeared to be of very low quality (total score 2 or 3). Confidence in ascertainment of exposure and of outcome assessment was very low in most studies. Even though most studies used a large sample of individual-level data, most data were collected by surveys that collected information using questionnaires on self-reported changes in several indicators on health-related behaviors as well as on consumption of medications. Some studies used aggregate data, which could mask individual-level effects, or self-reported questionnaires that had not been previously validated. Only six studies received a total score of >7, which was considered high quality.

### 3.1. The Financial Crisis and Lifestyle-Related Behaviors

#### 3.1.1. The Financial Crisis and Alcohol Consumption

Compared to the pre-crisis period, alcohol consumption decreased during or after among adults in Spain, Greece, and Italy [16,19,20,25,26,27,28,29,35], and among young Spanish people [13,43]. Two studies showed a significant upward overall trend in binge drinking [20]. Conversely, two studies provided evidence of increased prevalence of moderate [18] and heavy alcohol intake after the recession [16], while a Spanish study showed no differences [17].

When stratifying by socioeconomic level, controversial results were observed. Two studies suggested a widening of socioeconomic inequalities, although this effect is due to contrasting results: one study showed that heavy alcohol consumption increased during the crisis among the least educated men [16], while another showed an increase among the most educated people [17]. Finally, a more relevant reduction in alcohol intake among the lower class was also observed elsewhere, showing a narrowing of inequalities [25].

#### 3.1.2. The Financial Crisis and Smoking

Similarly to alcohol consumption, smoking habit also appeared to decrease markedly in most studies conducted in different settings or subgroups: young Spanish men aged 16-24 [13], adolescents in Portugal [21] and Spain [43], and adults in Greece [23,24] and Spain (only men) [25]. A reduction in smoking prevalence was observed in Spain also when stratifying by the size of the municipality [30], in a sample of patients hospitalized for cardiac catheterization [36], and in a sample of Spanish mothers [40]. A few studies reported a slight decrease [27,32], no difference [37], or a slight increase in the percentage of smokers after or during the crisis, for only women [16] in Spain and for both sexes in Italy [29,34], Portugal, and Spain [34].

Regarding socioeconomic status, controversial associations were reported during or after the crisis: a reduction in inequalities was identified by some studies, where percentages of daily or occasional smokers decreased more for those with a lower educational level [16], lower socioeconomic status [23], or having a manual occupation [40]. Conversely, other studies found an increase in socioeconomic inequalities due to an increase in smoking consumption among the unemployed [13,28,37,43] and among people belonging to a lower social class [25].

#### 3.1.3. The Financial Crisis and Healthy Diet

Generally speaking, a deterioration in the quality of diet was observed during and after the crisis. Several studies showed a reduction in the consumption of meat [16], fish [14,22], fruits [14,16,21,22,23,24,25], and vegetables [14,22,23,24] in Spain and Portugal, although in Portugal the consumption of vegetables increased among young people [21], and legumes were more frequently consumed [14,16]. Sweets and desserts were more often consumed by adults in Portugal [14] and Spain [16].

Several studies reported an increase in socioeconomic inequalities in healthy diet, especially regarding fruits and vegetables. In Spain, the probability of declaring eating fruit daily decreased more among unemployed men and least educated men, and the probability of declaring eating vegetables daily among unemployed men and women and the least educated women [16]. Two other Spanish studies reported an increase in inequalities in the consumption of fruits and vegetables [22,25].

Similar results were observed regarding fish consumption [22]. During the crisis in Italy, the socioeconomic differences in adherence to the Mediterranean diet widened, becoming less probable among people with a low wealth index score, those with a lower education level, and those performing manual labor [18]. One study conducted in Spain on young people below the age of 15 reported that the prevalence of junk food consumption increased in families with low maternal education level [33].

#### 3.1.4. The Financial Crisis and Physical Activity

Most of the studies highlighted an increase in physical activity in the adult population when comparing the periods after vs before the financial crisis, which was more robust and statistically significant in Spain [25,35] and Greece [23,24], and slighter in Portugal [21], while a limited reduction was observed in the few studies in adults [37] and in children [33,41].

Two Spanish studies reported an increase in socioeconomic inequalities in physical activity during and after the crisis. In one, an increase in the prevalence of physical activity was observed in all social classes, but this was slighter in the lower class, resulting in an increase in socioeconomic differences [25]. The other study observed that physical activity increased during or after the crisis among more educated women and decreased among the less educated, causing a widening of socioeconomic inequalities [16].

Instead, a Greek study showed a reduction in inequalities due to a significant increase in percentages of adults only, with those with a middle or lower socioeconomic status reporting high or moderate level of physical activity [24].

#### 3.1.5. The Financial Crisis and Use of Antidepressant, Anxiolytic, and/or Antipsychotic Drugs

Most studies reported an increase in any type of psychotropic drug use among all individuals considered [31,42] or only among women [16]. In a Spanish study performed on the economically active population, heavy use of hypnotics/sedatives among men and women increased in the period examined [20]. In a population study conducted in Portugal, the odds of consuming any psychotropic drug was estimated to be 1.5 times higher than before the crisis, and when evaluating the interaction effect of the year with sex and age, men and younger individuals reported higher odds of consuming any psychotropic drug [38]. Bartoll et al. [16] observed a stable trend in tranquilizer tablet use among men and a decrease among women.

This general increasing trend was not homogeneously reported for all psychotropic drugs: specifically, Marquez Calderon et al. [27] found an increase in sedative, tranquilizer, and hypnotic drugs, while antidepressant use decreased. According to Arroyo [15], only sedative use increased, and Madianos et al. evaluated only antidepressant use, finding an increase in its use in Greece [26].

When considering socioeconomic status, according to Arroyo et al. [15], the probability of consuming antidepressants or sedatives depended on employment status: in the case of individuals in short-term unemployment, both men and women showed that between 2006–2007 and 2011–2012, there was an increase in the risk of using sedatives. However, this increase was greater for women than for men. For the long-term unemployed, however, the differences between both sexes widened between 2006–2007 and 2011–2012: the risk of using sedatives in women increased, whereas it decreased in men, while antidepressant consumption decreased overall, and more markedly among short- and long-term unemployed subjects. Regarding education level, there was an increase in the intake of tranquilizers among those without any qualification and a slight decrease for men with high secondary education; among women, there was a drop-in intake, which was greater among those employed and those without any qualification [16].

Finally, an increase in psychotropic drug consumption was observed among households whose socioeconomic status was most affected by the crisis [42].

#### 3.1.6. The Financial Crisis and Substance Abuse

One of the included studies described a stable or a slight downward trend in drug use [28]. A study performed in Portugal on adolescents reported a slight decrease in monthly drug use [21]. A decrease in cocaine, marijuana, ecstasy, and hard drug use caused by the economic downturn, which could have affected the prices of these drugs, was observed by a Spanish study [28]. According to another Spanish study, overall cannabis use remained stable during the crisis, but unemployed men and women were more likely to have increased sporadic use compared to their employed counterparts [20]. Given a 10% increase in the provincial unemployment rate in Spain, an increase in the probability of using marijuana and cocaine in the last 30 days and also over the previous 12 months was observed [28].

## 4. Discussion

Since the financial crisis of 2008, southern European countries, in particular Italy, Greece, Spain, and Portugal, have been affected by the economic recession [44,45,46].

The research articles included in our review, published between 2011 and 2020, concerned the impact of the crisis on unhealthy behaviors, such as smoking habit [13,16,21,23,24,25,27,28,29,30,32], alcohol consumption [13,16,17,18,19,20,25,26,27,28,29,35,37,43], antidepressant/anxiolytic/antipsychotic use [15,16,20,26,27,31,38,42], and substance abuse [20,21,27,28] and on healthy behaviors, such as physical activity [16,21,23,24,25,27,30,33,35]. The studies on diet examined the impact of the crisis both on favorable and unfavorable eating behavior, a classification depending on the type of food considered for analyses [14,16,18,21,22,23,24,25,33,35,37].

According to the findings of our extensive review, we observed controversial effects on healthy behaviors in the period characterized by the 2008 financial crisis.

In general, the studies found that alcohol consumption [13,16,19,20,25,26,27,28,29,35,43] and substance abuse [21,28] decreased during or after the Great Recession, while psychotropic drug use increased [16,20,31,38,42].

A deterioration in proper eating habits was also observed [14,16,21,22,23,24,25], but some comments regarding diet are necessary. In fact, although most of the studies report an overall worsening of eating behavior during the crisis, the evaluation of its impact is more complex than it is for the other lifestyle-related behaviors. A reduction in the consumption of fish and meat, probably due to the reduction in available income, was observed, as was a reduction in fruit and vegetable consumption; both of these phenomena could cause a shift toward a worse diet. The crisis effect could have also contributed to determine the decrease in consumption of fruits and vegetables and the increase in junk food, sweets, and dessert consumption. Social inequality in proper eating habits generally increased because of the economic crisis [16,18,22,25,33]: several studies observed a decrease in the consumption of fruits and vegetables and an increase in junk food consumption among people with a low socioeconomic status.

However, even before the crisis, a decline in adherence to the Mediterranean diet had been observed in the younger population, so that a reduction in the mean consumption of fruit and vegetables was expected. Therefore, this decline may not be solely attributed to the economic crisis [23].

Most studies also showed an overall decrease in alcohol [13,16,19,20,25,26,27,28,35,37] and tobacco [13,21,23,24,25,27,32,34,35,36,37,40,43] consumption, while some highlighted an increase in tobacco consumption [16,29,34]. However, the pre- to post crisis variation in drinking and smoking habit were heterogeneous across socioeconomic levels, depending on the contexts and the dimension used to define socioeconomic status. For example, a study showed an increase in heavy alcohol consumption during the crisis among individuals with the lowest education level [16].

Less income available to purchase alcohol might have been behind the decrease in heavy drinking, while binge drinking could have increased as a means to deal with anxiety and emotional distress related to job loss, whether real or threatened, and to financial hardship [20]. However, it has been argued that a process of change in the pattern of alcohol use is taking place in Mediterranean countries such as Spain, where alcohol has traditionally been embedded in daily life, with wine drunk regularly with meals [20].

The decrease in tobacco use during times of economic downturn is related to price increases; it is likely that tobacco control measures may have interacted synergistically with the decline in disposable income. Therefore, austerity may have been a driving force in the decline among low-income individuals, along with the other public health measures [23].

Most of the studies found a slight increase in physical activity during the crisis [16,21,23,24,25,35], while other studies found a slight increase in sedentary habits [27,33,37,39,41]. There was an increase in socioeconomic differences in the prevalence of physical activity [16,25], with a heterogeneous gradient according to socioeconomic status.

The reduction in substance abuse observed during the crisis was slight, and generally referred to soft drugs [20,21,27,28], without any significant differences in terms of socioeconomic status.

Most studies reported an increase in the use of any psychotropic drug [20,26,27,31,38,42].

The decreased availability of income during the recession may have determined a reduction in the purchase of tobacco, alcohol, and drugs, but also of more expensive and healthier food. On the contrary, the increase in the use of psychotropic drugs can be considered a kind of coping mechanism against the insecurity and stress related to the economic crisis.

Our review appears to confirm previous evidence [47,48] that the financial crisis had an impact on socioeconomic inequalities and that negative effects on health tended to be more pronounced among the culturally, economically, and socially disadvantaged.

The low socioeconomic strata experienced inequalities in access to cultural and material resources (e.g., education, working conditions, income), which determined worse health and limited access to appropriate health care. These inequalities grew over the course of the global crisis, and the recession period could have accelerated the accumulation of such disadvantages [10,49,50].

The crisis itself may have played an independent, additional role, acting as a chronic stressor. Furthermore, it is possible that unemployed people and those at risk of unemployment or who experienced work instability may have had less time to dedicate to themselves and to their lifestyle, including food choice and physical activity [29].

In this scenario, the COVID-19 pandemic has exacerbated inequalities with a cumulative effect of the risks [51]. In England, as already shown in the Marmot Review, COVID-19 infection and mortality risks have been much higher for those living in more deprived areas, in overcrowded housing, in key workers in close proximity to others, in those from minority groups, in those with underlying health conditions, and in those who are older and/or male [52]. Furthermore, due to the effects of the mobility restrictions and the periods of lockdown as well as to the impoverishment of many sections of the population, it is reasonable to expect a further deterioration in lifestyles, especially among the people most affected by the economic crisis generated by the pandemic [53].

In fact, most countries were forced to introduce confinement measures to minimize the propagation of the SARS-CoV-2 virus, and for many people, it was difficult to maintain a healthy lifestyle, in particular a proper diet, regular physical exercise, quality of sleep, and limited smoking and alcohol consumption [53,54,55,56,57,58,59,60,61,62,63,64,65,66,67,68,69]. However, similar to the analysis reporting on the 2008 recession, the effects on diet are not unidirectional. In fact, many people used the period of home isolation to improve their eating habits and to limit dietary excesses and bad eating behaviors. Having the opportunity to devote time daily to having breakfast and to cooking meals resulted in an increase in the consumption of fruit, vegetables, and pulses [56,60,64,67,68], and a general decrease in alcohol consumption [56,66,67].

### Strengths and Limitations

Our review of the impact of the 2008 financial crisis on many health behaviors aimed to shed light on the links between changes in habits and health outcomes, an issue that has not yet been systematically investigated [3]. The focus on four southern European countries represents an added value, as they were hit harder by the crisis than were other European countries.

By focusing exclusively on health outcomes, our study did not look at the impact of the crisis on health systems, such as shortages in the health workforce or in medical supplies, for which several studies have shown a negative trend during the financial crisis [3,70,71].

The exact moment the financial crisis began is difficult to establish, and some studies, reporting data on different countries, have defined the duration of the crisis differently; this may have had an impact on the homogeneity of the reported results. The included studies had a high risk of bias in exposure and outcome assessment due to the study design, use of self-reported measures, and the lack of adjusting for potential confounding factors. Further, some observational studies did not apply any statistical tests [21,22,26,27,32,34,37,39,43]. Most importantly, although the studies included in our review investigated changes in population health status and health behaviors associated with the Great Recession, it cannot be established whether this was a causal relationship.

Due to the nature of the data of most of the included studies, which were not designed to measure exposure at the individual level, no causal relationship between the economic recession and changes in lifestyles can be established. We can, however, state that these changes occurred after the crisis. In fact, although a causal association between the financial crisis and trends in risk factors seems reasonable, we cannot exclude unmeasured confounding, which would provide alternative explanations for the observed trends. On the other hand, the financial crisis is a natural experiment at the population level and the possibility of its effects on the findings may be supposed [23].

Finally, self-reported information collected by questionnaire, as was the case for most of the included studies, may have been affected by information bias.

## 5. Conclusions

Our results seem to show that the crisis has had a negative effect on eating habits and a positive effect on alcohol consumption and on smoking, the consequence of mechanisms probably determined by decreased available income. Psychophysical stress linked to unemployment and job loss, as well as the worsening mental health observed in numerous studies, could explain the increase in the consumption of antidepressant and anxiolytic drugs.

These results suggest the need to implement health policies aimed at monitoring risk behaviors and for interventions aimed at contrasting the effects of the financial crisis in the countries studied. Inequalities in health behaviors should also be a priority area for action. Financial recommitment to public health system should accompany a substantial commitment to tackling the social determinants of poor health and wellbeing.

The identification of lifestyles and socioeconomic inequalities produced by the 2008 crisis may facilitate the understanding and the response to the possible effects of the current COVID-19 crisis.

The current pandemic provides harsh lessons on the societal vulnerabilities that arise from inequality. Investing in young people and supporting long-deprived regions and sectors of society are arguably the most powerful ways to break the chain of inequality transmitted from generation to generation. Adopting a broadened, equity-focused approach to population health should be an essential part of building a more resilient society that is better prepared to weather future pandemics.

## Figures and Tables

**Table 1:** Characteristics of the included studies by country.

COUNTRY: SPAIN				
Author	Study Design, Sample Characteristics		Outcome Definition	Findings *
**Aguilar-Palacio 2015** [13]	**Cross-sectional** Spanish National Health Survey (ENSE) Young people: 16–24 years N = 3701 (ENSE 2006: 2168; ENSE 2011/12: 1533)		**Alcohol consumption** in the last 2 weeks **Daily/occasional smoker**	**Alcohol consumption** Prevalence (%), 2006 vs. 2012 MEN: 61.9 vs. 56.3 (*p* = 0.015) WOMEN: 46.4 vs. 43.8 (ns) **Smoking** *Daily/occasional smokers* Prevalence (%), 2006 vs. 2012 MEN: 25.0 vs. 23.7 (ns) WOMEN: 28.9 vs. 21.9 (*p* < 0.001) Adjusted odds ratios (OR), 2012 vs. 2006: MEN: OR 0.97 (95% CI: 0.77–1.21) WOMEN: OR 0.79 (95% CI: 0.64–0.99) By socioeconomic status Adjusted odds ratios (OR), unemployed vs. working MEN 2006: OR 1.04 (95% CI: 0.64–1.69) 2012: OR 1.62 (95% CI: 1.00–2.62) WOMEN 2006: OR 0.97 (95% CI: 0.64–1.48) 2012: OR 1.24 (95% CI: 0.72–2.13)
**Arroyo 2019** [15]	**Cross-sectional** Adults: >15 years N = 49,463 (2006–2007: 28,954; 2011–2012: 20,509)		**Consumption of antidepressants or sedatives**	**Drugs** *Antidepressants* Overall consumption (%), 2006–2007 vs. 2011–2012: 5.8 vs. 4.3 (*p* < 0.01) *Sedatives* Overall consumption (%), 2006–2007 vs. 2011–2012: 10.7 vs. 11.1 (ns)
**Bartoll 2015** [16]	**Cross-sectional** Spanish National Health Survey (2001, 2003/04, 2006/07 and 2011/12) Economically active adults: 25–64 years N = 47,156		**Alcohol consumption** in the last 2 weeks **Heavy alcohol consumption** more than 17 Standard Basic Units of alcohol per week **Smoking habits** **Frequency of consumption of food** **Physical activity** moderate or intense physical activity **Tranquilizer or sleeping tablet intake** at least 1 tablet in the last 2 weeks.	**Alcohol** Trend percentages (%) of consumption, 2001–2012 Last two weeks: MEN: −5.4 (*p* < 0.01); WOMEN: −6.9 (*p* < 0.01) Heavy alcohol consumption: MEN: +2.0 (*p* < 0.01); WOMEN: −0.4 (ns) By socioeconomic status Adjusted regression coefficients (%) and p-value of the interaction between economic recession dummy (2001–2006/2007 vs. 2011/2012) and employment status (employed; unemployed) WOMEN Last two weeks −5.0; −12.4 (*p* = 0.054) Adjusted regression coefficients (%) and *p*-value of the interaction between economic recession dummy (2001–2006/2007 vs. 2011/2012) and education level (university, high secondary, lower secondary or primary, without any qualification) MEN: Heavy alcohol consumption +0.2; +0.8; +3.1; +5.4 (*p* = 0.012) WOMEN: Heavy alcohol consumption −1.5; +0.1; +1.4; −0.2 (*p* = 0.012) **Smoking** *Daily or occasional smokers* Trend percentages (%), 2001–2012 MEN: −0.8 (ns); WOMEN: +4.4 (*p* < 0.01) **Diet** Trend percentages (%) of consumption, 2001–2012 Fruits: MEN: −9.1 (*p* < 0.01); WOMEN: −7.9 (*p* < 0.01) Vegetables: MEN: −0.2 (ns); WOMEN: −2.4 (ns) Legumes: MEN: +3.4 (*p* < 0.05); WOMEN: +4.3 (*p* < 0.01) Meat: MEN: −9.7 (*p* < 0.01); WOMEN: −10 (*p* < 0.01) Cold meat: MEN: −4.7 (*p* < 0.01); WOMEN: −3.7 (*p* < 0.05) By socioeconomic status Adjusted regression coefficients (%) and *p*-value of the interaction between economic recession dummy (2001–2006/2007 vs. 2011/2012) and employment status (employed; unemployed) MEN: Vegetables daily: +9.0; 6.3 (*p* = 0.004) Fruits daily: −7.4; −12.1 (*p* = 0.041) Legumes (3 times or more per week): +3.8; −7.4 (*p* = 0.041) Fish (3 times or more per week): +1.2; −6.4 (*p* = 0.055) WOMEN Vegetables daily: −1.7; −4.3 (*p* = 0.065) Adjusted regression coefficients (%) and *p*-value of the interaction between economic recession dummy (2001–2006/2007 vs. 2011/2012) and education level (university, high secondary, lower secondary or primary, without any qualification) MEN: Fruits daily: −4.5; −6.1; −11.4; −21.8 (*p* = 0.06) Sweet food (3 times or more per week): −1.7; +2.5; +3.1; −16.9 (*p* = 0.067) WOMEN: Vegetables daily: −1.3; +3.4; −3.0; −27.1 (*p* = 0.004) **Physical activity** *Moderate or intense* Trend percentages (%), 2001–2012 MEN: +3.2 (*p* < 0.05); WOMEN: +1.4 (ns) By socioeconomic status Adjusted regression coefficients (%) and *p*-value of the interaction between economic recession dummy (2001–2006/2007 vs. 2011/2012) and education level (university, high secondary, lower secondary or primary, without any qualification) WOMEN: +6.5; +4.6; −1.6; −0.7 (*p* = 0.014) **Drugs** *Tranquilizers or sleeping pills* Trend percentages (%) of consumption, 2001–2012 MEN: −0.5 (ns); WOMEN: −2.4 (*p* < 0.01) By socioeconomic status Adjusted regression coefficients (%) and *p*-value of the interaction between economic recession dummy (2001–2006/2007 vs. 2011/2012) and employment status (employed; unemployed) WOMEN −3.7; +0.1 (*p* < 0.001) Adjusted regression coefficients (%) and *p*-value of the interaction between economic recession dummy (2001–2006/2007 vs. 2011/2012) and education level (university, high secondary, lower secondary or primary, without any qualification) WOMEN: −2.6; −1.2; −2.1; −18.3 (*p* = 0.051)
**Blázquez-Fernández 2019** [17]	**Cross-sectional** National Health Interview Survey Economically active adults: 18–65 years N = 29,677 (2006:14,696; 2011–2012: 14,981)		**Drinker** people consuming five or more drinks a week	**Alcohol** Prevalence (%), 2006 vs. 2011–2012 25.9 vs. 25.6 By socioeconomic status Adjusted OR: 2006 Unemployed less than 6 months: 0.87 (95% CI: 0.72–1.05) Education: Noncompulsory and pre-university secondary education: 0.90 (95% CI: 0.81–1.00) Specific labor training: 0.98 (95% CI: 0.85–1.14) University graduate: 0.85 (95% CI: 0.76–0.95) 2011–2012 Unemployed less than 6 months: 1.26 (95% CI: 1.06–1.50) Noncompulsory and pre-university secondary education: 1.36 (95% CI: 1.19–1.56) Specific labor training: 1.35 (95% CI: 1.13–1.61) University graduate: 1.37 (95% CI: 1.19–1.58)
**Colell 2015** [20]	**Cross-sectional** Economically active adults: 50–64 years N = 62,440		**Daily average of alcohol intake** in the last 30 days (grams of pure ethanol) **Heavy drinkers** above ≥ 40 g for men and ≥ 24 g for women **Binge drinking** 5 or more drinks on a single drinking occasion (within 2 h) at least once in the previous month in editions 2005 and 2007. Editions 2009 and 2011: 5 or more drinks for men and four or more for women **Hypnotics/sedatives** Sporadic users: use from 1 to 9 days in the last 30 days Heavy users: use from 10 to 30 days in the last 30 days	**Alcohol** *Daily average of alcohol intake (g/day), 2005–2007 vs. 2009*–*2011* MEN: 16.9 vs. 15.1 (*p* < 0.001) WOMEN: 7.7 vs. 7.1 (*p* = 0.002) *Heavy drinking* Prevalence (%), 2005–2007 vs. 2009–2011 MEN: 6.9 vs. 5.2 (*p* < 0.001) WOMEN: 3.3 vs. 2.8 (*p* = 0.013) Adjusted prevalence ratio (PR) (ref pre-crisis): MEN: 0.73 (95% CI: 0.67–0.79) WOMEN: 0.86 (95% CI: 0.75–0.99) *Binge drinking* Prevalence (%), 2005–2007 vs. 2009–2011 MEN: 19.3 vs. 22.0 (*p* < 0.001) WOMEN: 7.1 vs. 10.1 (*p* < 0.001) Adjusted prevalence ratio (PR) (ref pre-crisis): MEN: 1.17 (95% CI: 1.12–1.22) WOMEN: 1.62 (95% CI: 1.49–1.76) **Drugs** *Hypnotics/sedatives sporadic users* Prevalence (%), 2005–2007 vs. 2009–2011 MEN: 1.2 vs. 1.6 (*p* = 0.005) WOMEN: 2.1 vs. 2.4 (ns) *Hypnotics/sedatives heavy users* Prevalence (%), 2005–2007 vs. 2009–2011 MEN: 1.8 vs. 2.1 (*p* = 0.005) WOMEN: 3.7 vs. 5.4 (*p* < 0.001) Prevalence ratio (PR) (ref pre-crisis): MEN: 1.19 (95% CI: 0.99–1.42) WOMEN: 1.32 (95% CI: 1.17–1.49) By socioeconomic status Interaction (RRR) between activity (unemployed vs. employed) and period (2009–2011 vs. 2005–2007) MEN: 0.69 (95% CI: 0.49–0.97) **Substance abuse** *Cannabis sporadic users* Prevalence (%), 2005–2007 vs. 2009–2011 MEN: 5.6 vs. 5.2 (p ns) WOMEN: 3.1 vs. 2.4 (*p* < 0.001) Adjusted prevalence ratio (PR) (ref pre-crisis) MEN: 0.90 (95% CI: 0.81–1.01) WOMEN: 0.77 (95% CI: 0.64–0.91) By socioeconomic status Interaction (RRR) between activity (unemployed vs. employed) and period (2009–2011 vs. 2005–2007) MEN: 1.40 (95% CI: 1.10–1.77) WOMEN: 1.68 (95% CI: 1.17–2.41) *Cannabis heavy users* Prevalence (%), 2005–2007 vs. 2009–2011 MEN: 6.0 vs. 5.7 (ns) WOMEN: 2.0 vs. 1.9 (ns)
**Diaz-Mendez 2019** [22]	**Cross-sectional** Adults >16 years N = 50,485 (2006: 29,478; 2011–2012: 21,007)		**Frequency of consumption of food**	**Diet** Trend 2006–2011 Fruits (daily): falling Meat (3 or more times a week): rising Eggs (3 or more times a week): falling Fish (3 or more times a week): falling Pasta-rice-potatoes (daily): falling Bread (daily): remaining within guidelines. Vegetables (daily): falling Pulses (once or twice a week): rising Processed meats (occasionally/seldom or never): continuing Dairy (daily): falling Sweets (occasionally/seldom or never): falling Soft drinks (occasionally/seldom or never): continuing
**Garcia-Mayor 2019** [25]	**Cross-sectional** Spanish National Health Survey (SNHS) Adults 18–64 years N = 51,370 (2006: 28,478; 2012: 21,007; 2017: 23,089)		**Alcohol use** during the last 2 weeks **Tobacco use** **Fruit vegetable, pastries and/or sweets, sweetened beverages** daily intake (yes or no)	**Alcohol** Differences in prevalence, 2012 vs. 2006, 2017 vs. 2006 MEN: −4.7% (*p* < 0.001), −8.7% (*p* < 0.001) WOMEN: −3.3% (*p* < 0.001), −5.8% (*p* < 0.001) By socioeconomic status High (−2.8%, −4.4%) Middle (−1.2%, −4.0%) Low (−0.8%, −7.2%) **Smoking** Differences in prevalence, 2012 vs. 2006, 2017 vs. 2006 MEN: −3.5% (*p* < 0.001), −7.7% (*p* < 0.001) WOMEN: +0.3% (ns), −1.6% (ns) By socioeconomic status High (−3.4%, −7.0%) Middle (−0.4%, −5.2%) Low (−0.6%, −2.3%) **Diet** *Fruit consumption* Differences in prevalence, 2012 vs. 2006, 2017 vs. 2006 MEN: −3.9% (*p* < 0.001), −4.6% (*p* < 0.001) WOMEN: −7.2% (*p* < 0.001), −4.9% (*p* < 0.001) By socioeconomic status High (−5.2%, −1.2%) Middle (−5.4%, −5.3%) Low (−6.2%, −8.1%) *Vegetable consumption* Differences in prevalence, 2012 vs. 2006, 2017 vs. 2006 MEN: +4.8% (*p* < 0.001), −2.1% (*p* = 0.005) WOMEN: +3.5% (*p* < 0.001), −0.7% (ns) By socioeconomic status High (+4.6%, +2.3%) Middle (+5.2%, −1.0%) Low (+2.1%, −3.8%) *Sweets consumption* Differences in prevalence, 2012 vs. 2006, 2017 vs. 2006 MEN: −4.4% (*p* < 0.001), −8.8% (*p* < 0.001) WOMEN: −6.7% (*p* < 0.001), −9.3% (*p* < 0.001) By socioeconomic status High (−3.5%, −8.9%) Middle (−7.7%, −9.9%) Low (−5.4%, −9.2%) *Sweetened beverages* Differences in prevalence, 2012 vs. 2006, 2017 vs. 2006 MEN: −4.7% (*p* < 0.001), −3.5% (*p* < 0.001) WOMEN: −8.9% (*p* < 0.001), −5.0% (*p* < 0.001) By socioeconomic status High (−2.2%, −7.1%) Middle (−4.1%, −5.4%) Low (−5.2%, −8.1%) ** **Physical activity** Differences in prevalence, 2012 vs. 2006, 2017 vs. 2006 MEN: +0.7% (p: ns), +4.9% (*p* < 0.001) WOMEN: -4.7% (*p* < 0.001), +3.3% (*p* < 0.001) By socioeconomic status High (−0.1%, +7.6%) Middle (−0.3%, +6.3%) Low (−2.1%, +3.6%)
**Marquez-Calderon 2014** [27]	**Cross-sectional** Enquesta Domiciliaria sobre Alcohol y Droga en Espana (EDADES) Adults 15-64 years Enquesta Nacional de Salud de Espana (ENS)		**Alcohol consumption** In the last month (EDADES) Habitual (ENS) **Smoking habits** In the last month (EDADES) Daily (ENS) **Drugs use** In the last month: sedatives, tranquilizer, hypnotics (EDADES) Last 2 weeks: antidepressants (ENS)	**Alcohol** Prevalence (%), 2005 vs. 2011 (EDADES) and 2006 vs. 2012 (ENS) EDADES: 64.6 vs. 62.3; ENS: 48.4 vs. 38.3 **Smoking** Prevalence (%), 2005 vs. 2011 (EDADES) and 2006 vs. 2012 (ENS) EDADES: 38.4 vs. 37.6; ENS: 26.4 vs. 24.0 **Drugs** Prevalence (%), 2005 vs. 2011 Sedatives (EDADES): 3.7 vs. 8.3; Tranquilizers (EDADES): 2.7 vs. 6.9; Hypnotics (EDADES): 2.0 vs. 3.4; Antidepressants (ENS): 8.5 vs. 7.0 **Physical activity** *Sedentary lifestyle* Prevalence (%), 2006 vs. 2012: 39.4% vs. 41.3% **Substance abuse** Prevalence (%), 2005 vs. 2011 Cannabis: 8.7 vs. 7.0 Ecstasy: 0.6 vs. 0.3 Hallucinogens: 0.2 vs. 0.2 Amphetamines: 0.4 vs. 0.3 Cocaine powder: 1.6 vs. 1.1 Cocaine base: 0.1 vs. 0.1 Heroin: 0.1 vs. 0.1
**Martin Bassols 2016** [28]	**Cross-sectional** People aged 15-64 years N = 92,102 (2005: 27,400; 2007: 23,276; 2009: 19,713; 2011: 21,713)		**Alcohol consumption** **Smoking habits** **Substance abuse** marijuana and hard drugs such as crack, cocaine, heroin, ecstasy, hallucinogens, inhalants, and amphetamines in the last 12 months, last 30 days, every day in last 30 days	**Alcohol** Mean (%) 2005, 2007, 2009, 2011 Alcohol in last 12 months: 76.63, 72.92, 79,17, 77.08 Drunk in last 12 months: 22.01, 19.60, 27.16, 22.44 Alcohol in last 30 days: 63.24, 58.98, 62.48, 60.58 Alcohol every day in last 30 days: 12.05, 9.27, 9.31, 8.68 By socioeconomic status Probability (%) of consuming alcohol given a 10% increase in the provincial unemployment rate Alcohol in the past 12 months: −3.4 (*p* < 0.1) Not consuming any alcohol: +3 (*p* < 0.05) Consuming alcohol fewer than 20 days in the last year: 1.1 (*p* < 0.05) Consuming alcohol between 20 and 29 days during the last year: −0.1 (*p* < 0.05) Consuming alcohol between 30 and 150 during the last year: −1.7 (*p* < 0.05) Consuming alcohol more than 150 days during the last year: −2.3 (*p* < 0.05) **Smoking** Mean (%) 2005, 2007, 2009, 2011 Smoked in last 12 months: 31.81, 29.21, 31.78, 31.19 By socioeconomic status Probability (%) of smoking tobacco given a 10% increase in the provincial unemployment rate Smoked daily during the last 12 months: +3 (*p* < 0.01) **Substance abuse** *Drug consumption in last 12 months* Mean (%) 2005, 2007, 2009, 2011 Marijuana: 12.79, 10.56, 13.21, 11.38 Hard drugs: 4.03, 3.73, 3.99, 3.47 Cocaine: 3.31, 2.96, 3.09, 2.81 Ecstasy: 1.46, 1.17, 1.19, 0.91 *Drug consumption in last 30 days* Mean (%) 2005, 2007, 2009, 2011 Marijuana: 9.79, 7.42, 9.40, 8.26 Hard drugs: 2.21, 2.00, 1.93, 1.46 Cocaine: 1.76, 1.64, 1.43, 1.33 Ecstasy: 0.62, 0.41, 0.50, 0.33 By socioeconomic status Probability of using drugs in last 12 months given a 10% increase in the provincial unemployment rate Marijuana: +3.1 (*p* < 0.01) Hard drugs: +0.9 (ns) Cocaine: +1.2 (*p* < 0.01) Ecstasy: −0.4 (ns) Probability of using drugs in last 30 days given a 10% increase in the provincial unemployment rate Marijuana: +2.4 (*p* < 0.01) Hard drugs: +0.7 (ns) Cocaine: +0.9 (*p* < 0.1) Ecstasy: −0.2 (ns)
**Moreno Lostao 2019** [30]	**Cross-sectional** Spanish National Health Survey. People aged 15–74 years		**Tobacco consumption** daily and occasional smokers **Physical inactivity** no physical exercise and leisure time spent in sedentary habits	By socioeconomic status **Smoking** Age-adjusted percentage ratio by sex and area of residence (rural vs. large urban areas), 2006, 2011 and 2016 MEN: 0.99 (95% CI: 0.92–1.07), 0.90 (95% CI: 0.83–0.97), 0.89 (95% CI: 0.83–0.97) WOMEN: 1.09 (1.00–1.19), 0.96 (0.87–1.05), 1.03 (0.94–1.13) **Physical activity** Age-Adjusted percentage ratio of physical inactivity by sex and area of residence (rural vs. large urban areas), 2006, 2011 and 2016 MEN: 0.89 (95% CI: 0.86–0.92), 0.89 (95% CI: 0.83–0.95), 0.86 (95% CI: 0.79–0.92) WOMEN: 1.02 (95% CI: 0.98–1.06), 0.98 (95% CI: 0.91–1.03), 0.99 (95% CI: 0.92–1.05)
**Perez-Romero 2016** [31]	**Cross sectional** Spanish National Health Survey Adults aged 18-64 years N = 30,817 (2006–2007: 18,202; 2011–2012: 12,615)		**Drugs consumption** hypnotics and anxiolytics in the last 2 weeks	**Drugs** Adjusted odds ratios (OR), 2011-2012 vs. 2006–2007 MEN: OR 2.3 (95% CI: 1.8–2.8) WOMEN: OR 1.7 (95% CI: 1.4–1.9)
**Rajmil 2013** [33]	**Cross-sectional** Children <15 years old enrolled in Catalan Health Survey (ESCA) N = 4167 (2006: 2200; first wave 2010–2012: 1967)		**Junk food consumption** **Having breakfast at home** never vs. at least once per week **Physical Activity** **Time spent on screen**	**Diet** *Junk food consumption* Prevalence (%), 2006 vs. 2010–2012 50.24 (95% CI: 49.74–50.74) vs. 52.34 (95% CI: 51.92–52.76) By socioeconomic status Maternal education level: primary 47.46 vs. 50.14, secondary 50.21 vs. 52.13, university degree 52.79 vs. 53.78 Family employment status: employed 50.35 vs. 52.70, unemployed 50.25 vs. 51.04 *Never having breakfast* Prevalence (%), 2006 vs. 2010–2012: 4.9 (95% CI: 3.8–6.0) vs. 5.4 (4.8–6.7). By socioeconomic status Maternal education level: primary 5.9 vs. 7.7, secondary 5.1 vs. 6.4, university degree 3.5 vs. 2.7) Family employment status: employed 4.3 vs. 5.6, unemployed 8.8 vs. 4.4 **Physical activity** Prevalence (%), 2006 vs. 2010–2012 50.14 (95% CI: 49.52–50.76) vs. 48.23 (95% CI: 47.59–48.87) By socioeconomic status Maternal education level: primary 47.46 vs. 50.14, secondary 50.21 vs. 52.13, university degree 52.79 vs. 53.86 Family employment status: employed 50.35 vs. 52.70, unemployed 50.25 vs. 51.04 *Time (hours/day) spent on screen* Mean, 2006 vs. 2010–2012 2.03 (95% CI: 1.98–2.07) vs. 1.41 (95% CI: 1.35–1.47) By socioeconomic status Maternal education level: primary 2.16 vs. 1.73, secondary 2.08 vs. 1.53, university degree 1.77 vs. 1.07 Family employment status: employed 4.3 vs. 5.6, unemployed 8.8 vs. 4.4
**Regidor 2019** [35]	**Cross-sectional** Data taken from different sources		**Alcohol consumption** **Tobacco consumption** number of cigarettes sold per inhabitant aged ≥15 years; **Fruit and vegetable intake** measured by purchase **Physical activity** gone to a gym in the last 30 days, in population aged ≥15 years	**Alcohol** Annual percentage change (APC) in different time intervals: −0.1 (2004–2006) (ns) –2.3 (2008–2010) (*p* = 0.024) −0.2 (2011–2013) (ns) +2.1 (2014–2016) (*p* = 0.059) **Smoking** *Tobacco smoking* Annual percentage change (APC) in different time intervals: −1.9 (2004–2006) (ns) −8.3 (2008–2010) (*p* < 0.001) −13.5 (2011–2013) (*p* < 0.001) −1.1 (2014–2016) (ns) **Diet** *Fruit and vegetable consumption:* Annual percentage change (APC) in different time intervals: −0.1 (2004–2006) (ns) 2.1 (2008–2010) (*p* < 0.001) 1.2 (2011–2013) (*p* = 0.026) −1.9 (2014–2016) (*p* = 0.003) *Away-from-home dinners* Annual percentage change (APC) in different time intervals: −0.2 (2004–2006) (ns) –3.3 (2008–2010) (*p* < 0.001) −1.6 (2011–2013) (*p* = 0.009) 3.2 (2014–2016) (*p* < 0.001) **Physical activity** *Going to a gym* Annual percentage change (APC) in different time intervals: 0.2 (2004–2006) (ns) 4.4 (2008–2010) (*p* = 0.001) 1.6 (2011–2013) (ns) 7.1 (2014–2016) (*p* < 0.001)
**Spijker 2018** [39]	**Cross-sectional** Catalan Health Survey (ESCA) Adults aged >50 years N = 16,593 (2006: 6667; 2010–2012: 4458; 2013–2015: 5469)		**Sedentary life**	**Physical activity** *Sedentary life* Prevalence (%), 2006, 2010–2012, 2013–2015 MEN: 50–64 years old: 20.8, 18.3, 24.4 65+: 37.4, 28.0, 34.6 WOMEN: 50-64 years old: 18.9, 16.0, 22.5 65+: 41.7, 33.4, 41.9 By socioeconomic status Occupational status (employed, unemployed) Prevalence (%) 2006: 0.26, 0.31 2010–2012: 0.23, 0.26 2013–2015: 0.28, 0.34
**Trujillo-Aleman 2019** [40]	**Cross-sectional** N = 5919 mothers (2003–2004: 2951; 2011–2012: 2698)		**Smoking habits** daily and not daily smokers	**Smoking** Prevalence (%), 2003–2004 vs. 2011–2012 Couple mothers: 35.4 vs. 29.5 Lone mothers household heads: 42.7 vs. 39.1 Lone mothers-non household heads: 78.1 vs. 42.3 By socioeconomic status Social class (non-manual, manual) Lone mothers household heads vs. couple mothers Adjusted prevalence ratios, 2003-2004 2011-2012 Non-manual: 1.26 (95% CI: 1.00–1.59) 1.23 (95% CI: 0.95–1.58) p interaction ns Manual: 1.30 (95% CI: 1.08–1.57) 1.34 (95% CI: 1.08–1.66) p interaction ns
**Zapata Moya 2020** [42]	**Cross-sectional** People aged > = 18 N = 5679		**Anxiolytics and/or antidepressant consumption** before 2008 and in the last two weeks before the interview (2015)	**Drugs** Adjusted OR: 2015 vs. 2008 1.51 (95% CI: 1.05–2.42) By socioeconomic status Interaction between crisis impact on family SES and period (2015 vs. 2008) Adjusted OR: 2.18 (95% CI: 1.48–3.16)
**Zozaya 2020** [43]	**Cross sectional** Health Behavior in School-Aged Children (2002, 2006, 2010, 2014) Children and adolescents aged 9-21 years N = 77,651		**Alcohol consumption** drinking any alcoholic beverage at least every week **Smoking habit** frequent or occasional smoking during the last year	**Alcohol** Prevalence (%), 2002, 2006, 2010, 2014 17.57, 18.29, 16.09, 7.11 **Smoking** Prevalence (%) 2002, 2006, 2010, 2014 24.92, 16.10, 17.00, 10.00
**COUNTRY: ITALY**				
**Bonaccio 2014** [18]	**Cross-sectional** Moli-sani study Adults aged > 35 years N = 21,001	**Alcohol intake** **Adherence to Mediterranean diet (MD)** measured through the Italian Mediterranean Index score	**Alcohol** Mean grams/day of alcohol intake, 2005–2006 vs. 2007–2010 16.0 (SD: 21.8) vs. 16.9 (SD: 23.2) (*p* < 0.01) **Diet** % of high adherence to MD, 2005–2006 vs. 2007–2010 31.3 vs. 18.3 (*p* < 0.01) By socioeconomic status Prevalence ratios (PR), 2005–2006 and 2007–2010 High Wealth Index score vs. Low Wealth Index score: 1.05 (95% CI: 0.94–1.16) 1.31 (95% CI: 1.18–1.46) >13 years of education vs. < = 8 years of education: 1.16 (95% CI: 1.04–1.31) 1.32 (95% CI: 1.17–1.50) Manual non-manual job vs: 0.97 (95% CI: 0.82–1.15) 0.67 (95% CI: 0.57–0.79)	
**Mattei 2017** [29]	**Time trend analysis** People aged > = 15 years	**Alcohol consumption** **Smoking habit**	**Alcohol** *Overall rate of people aged 15 or more who consume alcohol more than once per week* Regression coefficient (beta), 2008–2015 vs. 2000–2007 −0.60 (95% CI: −0.96–−0.24) **Smoking** *Number of smokers* Regression coefficient (beta), 2008–2015 vs. 2000–2007 1.68 (95% CI: 0.17–3.20) *People who smoke more than 20 cigarettes per day* Regression coefficient (beta), 2008–2015 vs. 2000–2007 1.04 (95% CI: 0.45–1.62)	
**Petrelli 2017** [32]	**Cross-sectional** Istat National Health Interview Survey 2005 and 2013 Adults aged 18–64 years N = 153,137 (2005: 80,661; 2013: 72,476)	**Smoking habits**	**Smoking** *Current smokers* Prevalence (%), 2005–2013 Men: Italians 32.1–31.6, immigrants 33.7–32.5 Women: Italians 20.4–20.0, immigrants 17.5–16.2	
**Sarti 2018** [37]	**Time-trend analysis** Multipurpose surveys on aspects on daily life (2005–2013) Adults aged 30–64 years N = 187,731	**Alcohol consumption** at least 1/2 L per day of alcoholic drinks **Unbalanced diet** meat every day or fruit/vegetable consumption less than once a week or no vegetable oil use **Smoking habit** —current smokers —heavy smokers: > 20 cigarettes per day **Physical activity** Physical inactivity: no activity in the workplace, at home, or voluntary	**Alcohol** *Alcohol consumption* Prevalence (%), –2013 6.6, 6.4, 6.4, 5.7, 5.2, 4.9, 4.5, 3.9, 3.5 **Smoking** *Current smokers* Prevalence (%), –2013 25.9, 27.2, 26.6, 26.7, 27.5, 27.2, 26.6, 26.2, 25.2 *Heavy smokers* Prevalence (%), 2005–2013 8.6, 9.2, 8.8, 9.0, 8.8, 8.5, 8.1, 7.9, 7.0 **Diet** *Unbalanced diet* Prevalence (%), –2013 27.0, 26.6, 27.4, 27.5, 27.3, 26.8, 27.0, 26.0, 26.7 **Physical activity** *Physical inactivity* Prevalence (%), 2005–2013 13.3, 14.2, 13.7, 14.0, 14.1, 15.8, 14.5, 14.7, 14.5	
**COUNTRY: PORTUGAL**				
**Alves 2019** [14]	**Cross-sectional** National Health Interview Surveys Adults aged 25–79 years N = 43,273 (2005–2006: 41,193; 2014: 18,204)	**Diet** consumption of food groups in the day before the interview Fruits or vegetables: the number of days in the last week (2014)	**Diet** Prevalence (%) of consumption, 2005/2006 vs. 2014 fish 52 vs. 49 (*p* < 0.01) soup 68 vs. 64 (*p* < 0.01) fruit 82 vs. 73 (*p* < 0.01) vegetables 78 vs. 52 (*p* < 0.01) legumes 27 vs. 32 (*p* < 0.01) sweets/desserts 26 vs. 37 (*p* < 0.01)	
**de Matos 2015** [21]	**Time-trend analysis** Young people attending 6°, 8° and 10° year of school N = 15,953 (2014: 6026; 2010: 5050; 2006: 4877)	**Smoking habit** Tobacco consumption: —never —every day **Fruit or vegetable consumption** rarely or never; more than once per week **Physical activity** more than 3 times per week **Substance abuse** never, more than once in a month, hashish more than once.	**Smoking** Prevalence (%), 2006, 2010, 2014 never: 87.8, 88.1, 92.5 every day: 5.0, 4.5, 2.6 **Diet** *Rarely or never eats fruit* Prevalence (%), 2006, 2010, 2014 8.7, 7.7, 9.0 *Fruits more than once per week* Prevalence (%), 2006, 2010, 2014 22.2, 22.1, 21.2 *Rarely or never eats vegetables* Prevalence (%), 2006, 2010, 2014 12.2, 11.8, 14.6 *Vegetables more than once per week* Prevalence (%), 2006, 2010, 2014 11.7, 12.2, 13,3 **Physical activity (%)** Prevalence (%), 2006, 2010, 2014 46.7, 48.2, 51.0 **Substance abuse** Prevalence (%) of consumption, 2006, 2010, 2014 never: 95.5, 93.9, 96.7 more than once in one month: 2.6, 3.4, 2.1 hashish more than once: 8.2, 8.8, 8.8	
**Silva 2020** [38]	**Cross-sectional** World Mental Health Survey Initiative Portugal (2008/09) and the National Mental Health Survey Follow-Up (2015/16) Adults aged 18+ N = 911	**Drugs** use of psychotropic drugs in the previous 12 months	**Drugs** *Any psychotropic drug* Adjusted OR, 2015-16 vs. 2008-09 1.50 (95% CI:1.13–2.01) Interaction age*year: 18–49*2015–2016 1.95 (95% CI: 1.32–2.90) Interaction gender*year: men*2015–2016 1.85 (95% CI: 1.08–3.17) *Antidepressant* Interaction age*year: 18–49*2015–2016 1.68 (95% CI: 1.05–2.68) *Hypnotics/sedatives* 1.60 (95% CI: 1.14–2.25) Interaction age*year: 18–49*2015–2016 2.16 (95% CI: 1.34–3.47) Interaction gender*year: men*2015–2016 2.60 (95% CI: 1.36–4.98)	
**COUNTRY: GREECE**				
**Filippidis 2014** [23]	**Cross-sectional** Hellas Health I, II and IV Adults aged 18+ N = 3503 (2006: 1005; 2008: 1490; 2011: 1008)	**Current smokers** those who smoke every day or occasionally **Fruit and vegetable consumption** daily number of portions **Physical activity** high, moderate, or low	**Smoking** *Daily or occasional smokers* Difference 2011–2008–2006 (%): −11.56, *p* for linear trend: 0.014 By socioeconomic status Higher: −18.62, ns Middle: −1.06, ns Lower: −24.35, *p* = 0.023 **Diet** *At least 5 daily portions of fruit and vegetables* Difference 2011–2008–2006 (%): −66.27, *p* for linear trend: 0.001 By socioeconomic status Higher: −68.79, *p* < 0.001 Middle: −50.89, *p* < 0.001 Lower: −81.76, *p* < 0.001 **Physical activity** *High or moderate level of* *physical activity* Difference 2011–2008–2006 (%): +20.49 *p* for linear trend: 0.001 By socioeconomic status Higher: +10.66, ns Middle: +22.44, *p* = 0.001 Lower: +23.02, *p* < 0.001	
**Filippidis 2017** [24]	**Cross-sectional** Hellas Health I, II, III, IV, and V Adults aged 18+ N = 5504 (2006: 1005; 2008: 1490; 2010: 1000; 2011: 1008; 2015: 1001)	**Smoking habit** every day or occasionally **Fruit and vegetable consumption** daily number of portions **Physical activity** high, moderate, or low	**Smoking** *Current smokers* Prevalence (%), 2008 and 2015 42.6 (95% CI: 40.0−45.1) 36.5 (95% CI: 33.3−39.7) Adjusted risk ratio (RR) 2015 vs. 2008: 0.86 (95% CI: 0.77−0.95) **Diet** *Low fruit/vegetable consumption* Prevalence (%), 2008 and 2015 52.1 (95% CI: 49.6−54.7) 51.2 (95% CI: 47.9−54.6) Adjusted risk ratio (RR) 2015 vs. 2008: 1.00 (95% CI: 0.92−1.09) **Physical activity** *Sedentary lifestyle* Prevalence (%), 2006 and 2015 43.4 (95% CI: 40.2−46.6) 29.0 (95% CI: 26.0−32.0) Adjusted risk ratio (RR), 2015 vs. 2006: 0.69 (95% CI: 0.61−0.79)	
**Madianos 2014** [26]	**Time-trend analysis** Greek Population at census N = 10,387,000 (1991), 10,964,000 (2001), 10,939,000 (2011)	**Alcohol** per capita consumption (liters) **Antidepressant** consumption (daily unit)	**Alcohol** Per capita consumption (liters), 2005 and 2011: 9.24 and 8.80 **Drugs** Daily unit consumption of antidepressants, 2005 and 2011: 215.40, 284.85	
**Sanidas 2018** [36]	**Retrospective study** Hospitalized patients subjected to cardiac catheterization N = 3895 (2006-07: 1228; 2011-15: 2667)	**Current smokers** at least 1 cigarette per day	**Smoking** Prevalence (%) of smokers, 2006–2007 vs. 2011–2015: 45.4 vs. 36.9, *p* = < 0.001	
**Venetsanou 2020** [41]	**Cross-sectional** Children attending childcare centers (mean age: 52.72 ± 3.55 months) N = 652 (2009: 182; 2012: 161; 2015: 165; 2018: 144)	**Physical activity** measured with Omron Walking style pro HJ-720IT-E2 pedometer	**Physical activity** Step counts, 2009, 2012, 2015, 2018 Weekly: 8032 ± 2026, 7816 ± 2087, 6708 ± 2739, 6943 ± 2729 School-time: 3646 ± 1372, 3459 ± 1175, 3233 ± 1590, 2991 ± 1433 Leisure-time 4906 ± 1300, 4899 ± 1321, 4026 ± 1531, 4312 ± 1466 Weekend 6700 ± 2914, 7112 ± 2802, 5676 ± 3321, 6031 ± 3412 Statistically significant differences between cohort School-time: 2009 vs. 2018 Leisure-time: 2009 vs. 2015, 2009 vs. 2018, 2012 vs. 2015 Weekend: 2009 vs. 2015, 2012 vs. 2015	
**Country: Multicenter**				
**Bosque-Prous 2017** [19]	**Cross-sectional** Economically active adults: 50–64 years N = 25,479 (2006: 8016; 2013: 17463)	**Hazardous drinking** average daily consumption of >2 and >3 alcoholic drinks in the previous 3 months **Abstention** not drinking any alcoholic beverage during the 3 months prior to the interview	**Alcohol** *Hazardous drinking* Changes in the prevalence (%), 2006–2007 vs. 2013 SPAIN MEN: −5.4 (95% CI: −8.8–−2.0) WOMEN: −1.9 (95% CI: −5.7–−1.8) ITALY MEN: −5.6 (95% CI: −9.1–−2.2) WOMEN: −1.4 (95% CI: −4.7–−1.9) Adjusted prevalence ratio (PR), 2013 vs. 2006–2007 SPAIN MEN: PR = 0.42 (95% CI: 0.23–0.81) WOMEN: PR = 0.67 (95% CI: 0.24–1.97) ITALY MEN: PR = 0.44 (95% CI: 0.27–0.79) WOMEN: PR = 0.63 (95% CI: 0.21–1.70) *Abstention* Changes in the prevalence (%), 2006–2007 vs. 2013 SPAIN MEN: 3.3 (95% CI: −1.9–8.4) WOMEN: 3.5 (95% CI: −4.7–11.7) ITALY MEN: 6.8 (95% CI: 1.1–12.4) WOMEN: 5.2 (95% CI: −3.0–13.4) **Number of drinks per drinker per week** Crude relative risks (RR), 2013 vs. 2006–2007 SPAIN MEN: RR 0.59 (95% CI: 0.43–0.82) WOMEN: RR 0.63 (95% CI: 0.35–1.14) ITALY MEN: RR 0.65 (95% CI: 0.52–0.81) WOMEN: RR 0.60 (95% CI: 0.38–0.93)	
**Rathmann 2017** [34]	**Cross-sectional** Adolescents aged 15 Health Behaviour in School-aged Children (HBSC) study 2009–2010 N = 6554 (Greece *n* = 1606, Italy *n* = 1495, Portugal *n* = 1511, Spain *n* = 1942)	**Smoking habit** regular smokers: adolescents who smoke at least weekly	**Smoking** *Regular smokers* Prevalence (%) 2005–2006 vs. 2009–2010 Greece (16.2 vs. 15.2; −6.1%), Italy (19.8 vs. 22.3; +12.6%), Portugal (10.5 vs. 10.8; +3.1%), Spain (17.7 vs. 18.5; +4.6%)	

*: for stratified analysis, we report results of association when at least one strata is statistically significant.

**Table 2:** Variation of behavior influencing health status during or after the 2008 financial crisis.

First Author	Country	Alcohol	Smoking	Healthy Diet	Physical Activity	Drugs	Substance Abuse
Aguilar-Palacio 2015 [13]	Spain	−	−				
Arroyo 2018 [15]	Spain					−	
Bartoll 2015 [16]	Spain	−	+	−	+	−	
Blázquez-Fernández 2019 [17]	Spain	=					
Bosque-Prous 2017 [19]	Spain	−					
Colell 2015 [20]	Spain	−				+	−
Diaz-Mendez 2019 [22]	Spain			−			
Garcia Mayor 2020 [25]	Spain	−	−	−	+		
Marquez-Calderon 2014 [27]	Spain	−	−		−	+	−
Martin Bassols 2016 [28]	Spain	−	=				−
Moreno Lostao 2019 [30]	Spain		N/A		N/A		
Perez-Romero 2016 [31]	Spain					+	
Rajmil 2013 [33]	Spain			−	−		
Rathmann 2017 [34]	Spain		+				
Regidor 2019 [35]	Spain	−	−	+	+		
Spijker 2018 [39]	Spain				−		
Trujillo-Aleman 2019 [40]	Spain		−				
Zapata Moya 2020 [42]	Spain					+	
Zozaya 2020 [43]	Spain	−	−				
Bonaccio 2014 [18]	Italy	+		−			
Bosque-Prous 2017 [19]	Italy	−					
Mattei 2017 [29]	Italy	−	+				
Petrelli 2017 [32]	Italy		−				
Rathmann 2017 [34]	Italy		+				
Sarti 2018 [37]	Italy	−	−	=	−		
Alves 2019 [14]	Portugal			−			
de Matos 2015 [21]	Portugal		−	−	+		−
Rathmann 2017 [34]	Portugal		+				
Silva 2020 [38]	Portugal					+	
Filippidis 2014 [23]	Greece		−	−	+		
Filippidis 2017 [24]	Greece		−	=	+		
Madianos 2014 [26]	Greece	−				+	
Sanidas 2018 [36]	Greece		−				
Venetsanou 2020 [41]	Greece				−		
Rathmann 2017 [34]	Greece		−				

Note: statistically significant decrease (−); increase (+) or no variation (=) in the prevalence of healthy behavior; N/A: not applicable.

## Supplementary Materials

The following are available online at https://www.mdpi.com/article/10.3390/ijerph18168734/s1, Table S1: MEDLINE Search—Updated November 2020, Table S2: Risk of bias assessment for all the selected studies.
