# Supplementary material for: The 2008 Financial Crisis and Changes in Lifestyle-Related Behaviors in Italy, Greece, Spain, and Portugal: A Systematic Review
Source: Int J Environ Res Public Health. 2021 Aug 18;18(16):8734. doi: 10.3390/ijerph18168734 (PMC8392284; doi:10.3390/ijerph18168734)
Supplement: Supplementary file 1 [file ijerph-18-08734-s001.zip › ijerph-1308131-supplementary.pdf]

International Journal of
Environmental Research and Public Health

MDPI

Table S1. MEDLINE Search – Updated November 2020.

1 ((economic* or financial or banking or system) adj2 crisis).ab,ti.

2 (recession or austerity).ab,ti.

3 exp Economic Recession/

4 1 or 2 or 3

5 (health* or health?care).ab,ti.

6 (public adj2 care).ab,ti.

7 exp Public Policy/

8 unmet needs.ab,ti.

9 (mortality or death* or suicide* or depression).mp. [mp=title, abstract, original title, name of substance word, subject heading word, floating sub-heading word, keyword heading word, organism supplementary concept word, protocol supplementary concept word, rare disease supplementary concept word, unique identifier, synonyms]

10 (tobacco or smoking or cigarette* or alcohol or risk behavio* or exercise or phyisical activit* or healthy eating or healthy diet).mp. [mp=title, abstract, original title, name of substance word, subject heading word, floating sub-heading word, keyword heading word, organism supplementary concept word, protocol supplementary concept word, rare disease supplementary concept word, unique identifier, synonyms]

11 Substance-Related Disorders/

12 ((substance* or drug*) adj2 (abus* or addict* or depend* or misus* or use* or disorder* or consumption or overuse)).ab,ti.

13 (Prescription adj3 (diversion or misuse)).ab,ti.

14 5 or 6 or 7 or 8 or 9 or 10 or 11 or 12 or 13

15 4 and 14

16 (european or europe or Mediterranean or OECD or cross-country or cross-national or spain or italy or Portugal or Greece).tw.

17 (Spain or Italy or Portugal or Greece).sh.

18 16 or 17

19 15 and 18

20 limit 19 to yr="2008 -2020"

Table S2. Risk of bias assessment for all the selected studies.

Study	Selection	Comparability	Outcome	Total score
Aguilar-Palacio 2015	***	*	**	6
Alves 2019	****	**	**	7
Arroyo 2018	***	**	*	6
Bartoll 2015	***	**	**	7
Blázquez-Fernández 2019	**	**	**	6
Bonaccio 2014	***	**	**	7
Bosque 2017	***	**	**	7
Colell 2015	**	*	***	6
Diaz-Mendez 2019	**	**	**	6
Gaspar de Matos 2015	**		*	3
Filippidis 2014	**	**	**	6
Filippidis 2017	**	**	**	6
Garcia- Mayor 2020	***	**	**	7

Madianos 2014	***	*	**	6
Marquez-Calderon 2014	**	-	*	3
MartinBassols 2016	***	*	**	6
Mattei 2017	**		**	4
Moreno-Lostao 2019	***	*	**	6
Perez -Romero 2016	**	*	**	5
Petrelli 2016	**	*	**	5
Rajmil 2013	**	*	**	5
Rathmann 2017	**	.	**	4
Regidor 2019	**	-	**	4
Sanidas 2018	*	-	*	2
Sarti 2018	**	-	*	3
Silva 2020	**	*	**	5
Spijker 2018	***	-	**	5
Trujillo-Alemán 2019	***	*	**	6
Venetsanou 2020	**	-	*	3
Zozaya 2020	***	**	**	7
Zapata Moya 2020	**	**	**	6
